# The Monkey game: A computerized verbal working memory task for self-reliant administration in primary school children

**DOI:** 10.3758/s13428-015-0607-y

**Published:** 2015-06-20

**Authors:** Eva Van de Weijer-Bergsma, Evelyn H. Kroesbergen, Shahab Jolani, Johannes E. H. Van Luit

**Affiliations:** Department of Pedagogical and Educational Sciences, Utrecht University, P.O. Box 80140, 3508 TC Utrecht, The Netherlands; Department of Methodology and Statistics, Utrecht University, Utrecht, The Netherlands

**Keywords:** Verbal working memory, Psychometric properties, Children, Computerized assessment

## Abstract

In two studies, the psychometric properties of an online self-reliant verbal working memory task (the Monkey game) for primary school children (6–12 years of age) were examined. In Study 1, children (*n* = 5,203) from 31 primary schools participated. The participants completed computerized verbal and visual–spatial working memory tasks (i.e., the Monkey game and the Lion game) and a paper-and-pencil version of Raven’s Standard Progressive Matrices. Reading comprehension and math achievement test scores were obtained from the schools. First, the internal consistency of the Monkey game was examined. Second, multilevel modeling was used to examine the effects of classroom membership. Multilevel multivariate regression analysis was used to examine the Monkey game’s concurrent relationship with the Lion game and its predictive relationships with reading comprehension and math achievement. Also, age-related differences in performance were examined. In Study 2, the concurrent relationships between the Monkey game and two tester-led computerized working memory tasks were further examined (*n* = 140). Also, the 1- and 2-year stability of the Monkey game was investigated. The Monkey game showed excellent internal consistency, good concurrent relationships with the other working memory measures, and significant age differences in performance. Performance on the Monkey game was also predictive of subsequent reading comprehension and mathematics performance, even after controlling for individual differences in intelligence. Performance on the Monkey game was influenced by classroom membership. The Monkey game is a reliable and suitable instrument for the online computerized and self-reliant assessment of verbal working memory in primary school children.

Working memory is the ability to temporarily store and manipulate information simultaneously and is considered an important predictor for academic performance in areas such as reading (De Weerdt, Desoete, & Roeyers, 2013; Gathercole, Alloway, Willis, & Adams, 2006; Swanson, Xinhua, & Jerman, 2009) and mathematics (Bull, Espy, & Wiebe, 2008; Friso-van den Bos, Van der Ven, Kroesbergen, & Van Luit, 2013; Geary, Hoard, Byrd-Craven, Nugent, & Numtee, 2007; Swanson, 2006; Swanson, Jerman, & Zheng, 2008; Toll, Van der Ven, Kroesbergen, & Van Luit, 2011). Children who are better able to hold relevant information in mind and manipulate this information have an advantage in integrating information from different passages while reading a written text, as well as an advantage in choosing and carrying out strategies while solving math problems. Working memory in children is usually assessed with computer- or paper-and-pencil tests in a one-to-one testing situation led by a test assistant, which is very time-consuming and costly. Computerized (online) working memory tests that can be administered self-reliantly or in groups could be of great value for studying working memory in large-sample studies. The aim of this study was to investigate the psychometric properties of a verbal working memory task for self-reliant (group) administration in primary school children.

Working memory is generally viewed as a multicomponent system, in which domain-specific storage and rehearsal components or processes interact with a domain-general attentional control component (Baddeley, 2000; Baddeley & Hitch, 1974; Engle, 2002; Engle, Tuholski, Laughlin, & Conway, 1999; Kane, Hambrick, Tuholski, Wilhelm, Payne, & Engle, 2004). Baddeley’s model of working memory, for example—which is the most frequently referred-to model—includes the central executive, phonological loop, visuospatial sketchpad, and episodic buffer (Baddeley, 2000; Baddeley & Hitch, 1974). The central executive is a domain-general attentional control system involved in several processes, such as the selection and execution of strategies, monitoring of input, retrieval of information from long-term memory, storing and processing of information, and coordination of the other components of the working memory system. The two domain-specific slave systems, the phonological loop and the visuospatial sketchpad, involve the temporary storage and rehearsal of phonological and auditory information and visual and spatial information, respectively. The episodic buffer—a temporary storage system that is responsible for the integration of information from a variety of sources—is the third slave system (Baddeley, 2000). The functioning of the phonological loop and the visuospatial sketchpad is typically measured using simple span tasks, in which increasingly longer strings of information are immediately recalled without further processing. The functioning of the central executive is usually measured with complex span tasks, requiring the storage as well as the processing or manipulation of information (Kail & Hall, 2001). In other words, working memory can be distinguished from short-term memory, which only involves the temporary storage of information by the slave systems, whereas working memory involves the storage as well as processing of information. Although the central executive is a domain-general component of working memory, the tasks used to assess its functioning also tap into one (or both) of the domain-specific slave systems. According to Engle and colleagues (Engle, 2002; Engle et al., 1999; Kane et al., 2004), working memory capacity is mainly determined by the domain-general executive component. Using a latent-variable approach with an adult sample, Kane et al. (2004) found that a two-factor working memory model with separable verbal and spatial factors provided the best fit. However, the large proportion of variance shared between the two factors (70 %) indicated that performance on working memory tasks is primarily determined by a domain-general mechanism. So, even though the domain-specific storage components also play roles, the shared variance between measures of working memory primarily reflects the contribution of the domain-general executive component according to this view. Nevertheless, the multicomponent nature of these working memory models allows researchers to examine whether the contributions of different subcomponents vary as a function of, for example, age or the academic domain investigated.

Although the central executive component of working memory is domain-general, reading comprehension seems to be more strongly related to verbal than to visual–spatial working memory tasks (Seigneuric, Ehrlich, Oakhill, & Yuill, 2000). In order to comprehend a written text as a coherent whole, children have to integrate neighboring passages and need to make inferences about different events, actions, and states. Verbal working memory allows children to hold the most recently read propositions in mind while establishing coherence, and also allows children to retrieve information (e.g., from the text or prior knowledge) from long-term memory for integration with the current text (Cain, Oakhill, & Bryant, 2004). Although visual–spatial working memory tasks also have been found to be related to reading, Savage, Lavers, and Pillay (2007) argued in their review of the literature that previous findings of visual–spatial working memory deficits in reading problems are probably the result of sampling issues (e.g., the comorbidity of reading difficulties and other learning difficulties; Savage et al., 2007). Indeed, a recent meta-analysis indicated that deficits in the phonological loop and central executive most prominently underlie reading difficulties in children with average intelligence (Swanson et al., 2009). Mathematics performance and learning, on the other hand, has been shown to be related to performance on both visual–spatial and verbal working memory tasks (De Smedt, Janssen, Bouwens, Verschaffel, Boets, & Ghesquière, 2009; Friso-van den Bos et al., 2013; Imbo & Vandierendonck, 2007; Swanson, 2006; Toll et al., 2011; Van der Ven, Van der Maas, Straatemeier, & Jansen, 2013). Solving mathematical problems may elicit visual–spatial as well as verbal representations and strategies, which vary with age and the type of math test that is used (Friso-van den Bos et al., 2013; Raghubar, Barnes, & Hecht, 2010). In sum, whereas performance on visual–spatial working memory tasks is mainly related to mathematics achievement, performance on verbal working memory tasks seems to be related to both mathematics achievement and reading comprehension (Geary, 2011).

A domain-general ability that is strongly related to working memory and also influences academic achievement is intelligence (Geary, 2011). Despite the finding that working memory measures and measures of intelligence share substantial variance, they are considered distinct constructs (Conway, Cowan, Bunting, Therriault, & Minkoff, 2002; Engle et al., 1999; Kane et al., 2004).

## Online working memory assessment

Although working memory can be assessed with a wide variety of measures, standardized performance-based tests (e.g., paper-and-pencil or computerized) give a more objective representation of the differences between individuals than do behavioral ratings (Alloway, Gathercole, Kirkwood, & Elliott, 2009; Mahone et al., 2002; Mangeot, Armstrong, Colvin, Yeates, & Taylor, 2002; Toplak, Bucciarelli, Jain, & Tannock, 2008; Vriezen & Pigott, 2002). Since standardized performance-based test assessment in a one-to-one testing situation is very time-consuming and costly, a growing number of computerized (online) working memory tests can be administered self-reliantly or in groups. Although researchers have less control over behavioral assessment when using self-reliant online assessment, online assessment also provides researchers the possibility to collect data in much larger samples than in more controlled settings (Van de Weijer-Bergsma, Kroesbergen, Prast, & Van Luit, 2014; Van der Ven et al., 2013). The feasibility of computerized or online self-reliant working memory tasks has been shown in adults (De Neys, d’Ydewalle, Schaeken, & Vos, 2002; Pardo-Vázquez & Fernández-Rey, 2008), as well as in primary school children (Van de Weijer-Bergsma et al., 2014; Van der Ven et al., 2013). To the best of our knowledge, previous studies with primary school children have focused on visual–spatial working memory tasks (Van de Weijer-Bergsma et al., 2014; Van der Ven et al., 2013), whereas self-reliant assessment of verbal working memory tasks has not yet been examined.

## Assessment of verbal working memory in children

Verbal working memory in children is usually assessed using complex span tasks (e.g., reading span, counting recall, listening recall), *n*-back tasks (e.g., letter memory), or backward span tasks (e.g., digit span backward, word span backward).

In complex span tasks, participants have to perform a task—for example, reading sentences and verifying their logical accuracy while remembering the last word from each sentence within a set, or counting objects and remembering the total in each set of objects. After the set of items (e.g., sentences or counts) within one trial is finished, recall of the remembered words or total counts is prompted. The task difficulty is increased by increasing the number of items within one set. Obviously, because young children are only starting to learn how to read from the beginning of primary school, sentence-reading tasks are not very suitable for children in early primary school.

In *n*-back tasks, participants are presented with a varying number of stimuli (e.g., letters, words) and are asked to recognize whether the last stimulus was the same as the one presented *n* stimuli previously (where *n* is usually 1, 2, or 3), or are asked to recall the last *n* (e.g., 4) words of the list after a list has ended. Such tasks require participants to update the last *n* stimuli, since the participants do not know when a set will end. It should be noted that the concurrent validity of *n*-back tasks using recognition with other working memory measures has not been shown in adults (Jaeggi, Buschkuehl, Perrig, & Meier, 2010; Kane, Conway, Miura, & Colflesh, 2007), and is therefore questioned. *N*-back tasks using recall have been found to show stronger associations with complex span tasks (Shelton, Elliott, Matthews, Hill, & Gouvier, 2010; Shelton, Metzger, & Elliott, 2007).

Backward span tasks generally begin with a short list of verbal items to remember, with the number of items increasing over successive trials in order to increase working memory load. After the items within a given set are presented, the participant is prompted to recall the items in a backward fashion. Although some studies with adult samples have suggested that backward span tasks are measures of short-term memory, rather than working memory, since they only require a mental transformation of the order of the verbal materials (Cantor, Engle, & Hamilton, 1991; Engle et al., 1999), others have found that mere sequence transformation could be sufficient to tap into working memory (Oberauer, Süß, Schulze, Wilhelm, & Wittmann, 2000). Moreover, in children, even simple span tasks may require more executive processing, since sequencing the order of items may be less proceduralized in children (Hutton & Towse, 2001). Several studies have shown that backward span tasks require executive processing in children, and can therefore be considered a measure of working memory during childhood (Alloway, Gathercole, & Pickering, 2006; Gathercole, Brown, & Pickering, 2003).

## Development and characteristics of the Monkey game

**Fig. 1:**
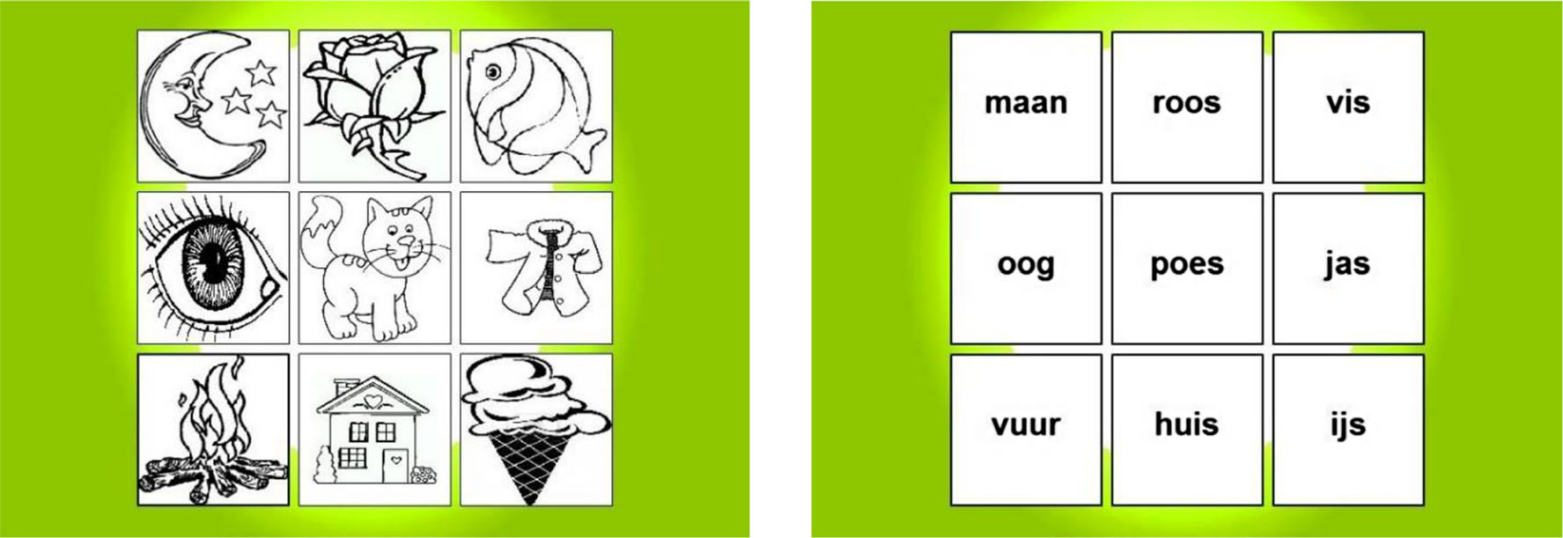
Screenshots of the Monkey game response formats for Grade 1 (left) and Grades 2–6 (right; in Dutch)

The Monkey game is a backward word span task. The theme “Monkey game” was chosen because of the inclination of monkeys to copy or imitate behavior. During the introduction of the game (but not during the instructions) and after completion of each level, different animations of cartoon monkeys are shown to emphasize the theme of the task and sustain children’s engagement. Children hear a number of spoken words, which they have to remember and recall backward by clicking on the words presented visually in a 3 × 3 matrix. The nine words that are used in the task (i.e., *moon*, *fish*, *rose*, *eye*, *house*, *ice*, *fire*, *cat*, and *coat*) are some of the words first learned to read by Dutch children in first grade (i.e., *maan*, *vis*, *roos*, *oog*, *huis*, *ijs*, *vuur*, *poes*, and *jas*), and reading these single words is therefore assumed to be automatized in most second-grade students. These words were chosen to minimize the influence of differences in reading ability. Unlike most backward word span tasks, the Monkey game does not require participants to give a verbal answer, but instead requires a response in the visual–spatial format of a 3 × 3 matrix. Children from Grade 1 are presented with pictures of the words, since word identification may not have yet have been automatized by children in this grade. Children from Grades 2–6 are presented with written words (see Fig. 1 for screenshots of the game response format). The task consists of five levels, in which working memory load is manipulated by the number of words that children have to remember and recall backward, ranging from two in Level 1 to six in Level 5. Item sets were constructed using randomization with regard to the sequence of words. Before starting the task, all children are presented with four practice sets. In the first two practice sets, children are asked to recall two words forward. After those sets, children are informed about the backward recall procedure and are presented with two more practice sets in a backward fashion. After each practice set, children receive feedback on their performance. Then the assessment starts. Within each set, an item is scored as correct only if it was recalled in the correct serial position. The number of correct items within each set is then converted into a proportion correct score, by dividing it by the total number of items within that set. So, in the trial *moon*, *rose*, *fish*, *house*, for example, the answer *house*, *rose*, *fish*, *moon* would have resulted in a .50 proportion correct score for that trial, since *house* and *moon* are recalled at the correct backward serial positions. Then the mean proportion scores recalled over all sets are calculated and used as the outcome measure (St Clair-Thompson & Sykes, 2010), with scores ranging from 0 to 1.

## Study 1

The goal of this study was to examine whether the Monkey game can be used as a self-reliant online computerized measure for verbal working memory. The quality of the task was assessed by examining the internal consistency of the different items, the relationship between performance on the Monkey game and age (i.e., to see whether the task reflects the development of working memory during childhood), its concurrent relationship with a self-reliant computerized visual–spatial working memory task (concurrent validity), and its predictive relationships with achievement scores on mathematics and reading comprehension tests (predictive validity). These relationships were examined before and after controlling for intelligence. Although controlling for intelligence might remove important shared variance between working memory and intelligence, it might also strengthen evidence for an unique contribution of working memory to academic achievement. Also, we explored whether the concurrent and predictive validity of the Monkey game is influenced by age.

First, we expected older children to perform better on the Monkey game than younger children (Alloway & Alloway, 2010; Alloway et al., 2006; Van der Ven et al., 2013). Second, since both verbal and visual–spatial working memory tasks tap into domain-general executive processing, we expected that Monkey game performance would be related to performance on the visual–spatial working memory task (i.e., the Lion game; see the Measures section) (Engle, 2002; Engle et al., 1999; Kane et al., 2004). Third, since verbal working memory is more important for reading comprehension than is visual–spatial working memory (Seigneuric et al., 2000; Swanson et al., 2009), we expected the Monkey game to be a stronger predictor of reading comprehension than the Lion game. Fourth, we expected both the Monkey game and the Lion game to be predictors of math achievement (De Smedt et al., 2009; Friso-van den Bos et al., 2013; Imbo & Vandierendonck, 2007; Swanson, 2006). Fifth, we expected these relationships to remain after controlling for intelligence (Conway et al., 2002; Engle et al., 1999; Kane et al., 2004).

### Method

#### Participants

The data used in this study are part of a large-scale intervention study of the effects of teacher training in differentiated math education on student math performance.^1^ A total of 5,203 children participated from Grades 1 to 6 from 31 elementary school in the Netherlands. The schools (and their student populations) can be viewed as very diverse and as a good representation of the schools and students in the Netherlands, since they were located in different parts of the country (e.g., rural as well as urban areas) and in different neighborhoods (e.g., lower- as well as middle-class neighborhoods), and had different school sizes (ranging from 52 to 550 students). The parents of all children received written information about the study and a passive informed consent procedure was used in which parents informed their child’s teacher or a designated contact person at their school if they did not want their child to participate. The study was approved by the ethics committee of the Faculty of Social and Behavioral Science, Utrecht University.

#### Measures

##### Working memory

Two online computerized working memory tasks were administered, the Monkey game and the Lion game. For a description of the Monkey game, see the Development and Characteristics of the Monkey Game section.

The Lion game is a visual–spatial complex span task, in which children have to search for colored lions (Van de Weijer-Bergsma et al., 2014). Children are presented with a 4 × 4 matrix containing 16 bushes. In each set, eight lions of different colors (red, blue, green, yellow, and purple) are consecutively presented at different locations in the matrix for 2,000 ms each. Children have to remember the *last* location where a lion of a certain color (e.g., red) appeared and use the mouse button to click on that location after the sequence has ended. The task consists of five levels in which working memory load is manipulated by the number of colors—hence, the number of locations—that children have to remember and update. Within each set, an item was scored as correct only if it was recalled in the correct serial position. The number of correct items within each set was then converted into a proportion correct score by dividing it by the total number of items within that set. Then the mean proportion scores recalled over all sets were calculated and used as an outcome measure (St Clair-Thompson & Sykes, 2010), with scores ranging from 0 to 1. The Lion game has shown excellent internal consistency (*α* ranges from .86 to .90), satisfactory 6-week test–retest reliability, and good concurrent relationships with individually administered (tester-led) working memory tasks, and it is predictive of subsequent mathematics performance (Van de Weijer-Bergsma et al., 2014).

##### Intelligence

The Raven Standard Progressive Matrices (SPM) were used (Raven, Court, & Raven, 1996) as a measure of nonverbal intelligence. The Raven SPM consists of five series (A to E) of 12 diagrams or designs with one part missing. Children are asked to select the correct part to complete the designs from among six (series A and B) or eight (series C to E) answer options printed beneath. Children have to decide which of the alternatives given logically completes the design. The test starts relatively easy but increases in difficulty, and answers are scored as incorrect (0) or correct (1). The minimum score is 0 and the maximum score is 60. Internal consistency was *α* = .92 in this study.

##### Reading comprehension

Reading comprehension was measured using the criterion-based Cito Reading Comprehension Tests (Feenstra, Kamphuis, Kleintjes, & Krom, 2010; Weekers, Groenen, Kleintjes, & Feenstra, 2011). The reading comprehension tests are national Dutch tests used to monitor the progress of primary school children. For Grades 1–6, seven tests are used, normed for different periods within grades (B = beginning of the school year, M = mid-school-year, E = end of school year): E1, M2, E2, M3, M4, M5, B6, and M6. The test consists of three modules that allow for differentiated testing. All children start with the Start module, and on the basis of their performance, children finish the module Sequel 1 (for lower-performing students) or Sequel 2 (for higher-performing students). The tests consist of different reading passages, followed by a total of 50 (Grades 1–4) or 55 (Grades 5 and 6) multiple-choice questions. The raw scores are converted into ability scores that increase throughout primary school, enabling comparison of the results from different versions. Ability scores vary from –87 (lowest in Grade 1) to 147 (highest in Grade 6). Validity and reliability have been reported as satisfactory (Cronbach *α* ranges from .84 to .93; Feenstra et al., 2010).

##### Mathematics performance

Similar to the reading comprehension tests, the criterion-based Cito Mathematics Tests are national Dutch tests used to monitor the progress of primary school children (Janssen, Scheltens, & Kraemer, 2005). These tests primarily consist of contextual math problems. There are two different versions for each grade, one to be administered at mid-school-year (M) and one at the end of the school year (E), except for Grade 6, which has a test at the beginning of the school year (B6) and one at mid-school-year (M6). In each test, five main domains are covered: (a) numbers and number relations; (b) addition and subtraction; (c) multiplication and division; (d) complex math applications, often involving multiple mathematical manipulations; and (e) measuring (e.g., weight and length). From M2 through M6, several domains are added successively: (f) estimation, (g) time, (h) money, (i) proportions, (j) division, and (k) percentages. The raw test scores are converted to ability scores that increase throughout primary school, enabling comparison of the results from different tests on the same scale (Janssen et al., 2005). Ability scores vary between 0 (lowest in Grade 1) and 169 (highest in Grade 6). The reliability coefficients of the different versions range from .91 to .97 (Janssen, Verhelst, Engelen, & Scheltens, 2010).

#### Procedure

Measurements took place on two occasions during the school year of 2012–2013, in September–October 2012 (T1) and January–February 2013 (T2). At T1, visual–spatial working memory was assessed using the Lion game. Teachers received an e-mail containing login information for their class of children and were asked to let all students within their class finish the task within a period of 3 weeks. Also at T1, a paper-and-pencil version of Raven’s SPM was administered in classrooms by research assistants as a group test. Assessment was stopped after 60 min, even if some of the children did not finish the test. At T2, verbal working memory was assessed with the Monkey game, using a procedure identical to that for the Lion game. Mathematics performance and reading comprehension tests were administered as part of the regular school testing procedure, and the results were requested from the mid-school-year results (January–February 2013, around T2).

#### Data analysis

In large samples, a few outliers are to be expected (Tabachnick & Fidell, 2007). Five univariate outliers were detected (*Z* scores > 3.29) in the reading comprehension scores. These were high but realistic scores in Grades 5 and 6, and were therefore not removed. Fifteen potential multivariate outliers were detected on the basis of Mahalanobis distances [*χ*^2^(3) = 16.27]. However, the influence of these outliers was negligible (Cook’s distance < 0.13). Therefore, the values were not deleted or transformed. The normality of the distributions of variables was examined by calculating the standardized skewness and kurtosis indices (statistic divided by standard error). The skewness index was found to be higher than 3 for the Raven SPM (–10.51), whereas both the skewness and kurtosis indices were found to be higher than 3 for the Monkey game (–17.82 and 4.49, respectively), the Lion game (–23.24 and 3.88, respectively), mathematics performance (–8.17 and –10.28, respectively), and reading comprehension (9.64 and 7.05, respectively), indicating that the distributions differed significantly from normality. Nonnormality was therefore taken into account in all statistical analyses.

The reliability of the Monkey game was determined by calculating the internal consistency of the items in two different ways for the whole sample and for the different grades separately. First, we calculated the sum of the proportion correct scores for the first trials of the different levels (i.e., Trial 1 of Level 1, Trial 1 of Level 2, etc.) and calculated the same for the second, third, and fourth trials of different levels. Then Cronbach’s alpha was calculated between these scores (Engle et al., 1999). Second, Cronbach’s alpha was calculated for the proportion correct scores on each individual trial (Kane et al., 2004).

To examine whether classroom membership influences performance on the Monkey game, we analyzed the ratio of variance *between* classes in Monkey game performance to variance *within* those classes, using grade as a control variable. The intraclass correlation (ICC) was analyzed using a multilevel analysis with a two-level structure (Level 1, individual children; Level 2, class) with Mplus version 7.0 (Muthén & Muthén, 2006). ICC values of .05, .10, and .15 were considered to be small, medium, and large, respectively (Hox, 2002). Also, a design effect was calculated as 1 + (average cluster size – 1) × ICC (Muthén & Satorra, 1995). A design effect greater than 2 would indicate that clustering in the data needed to be taken into account during the estimation.

**Fig. 2:**
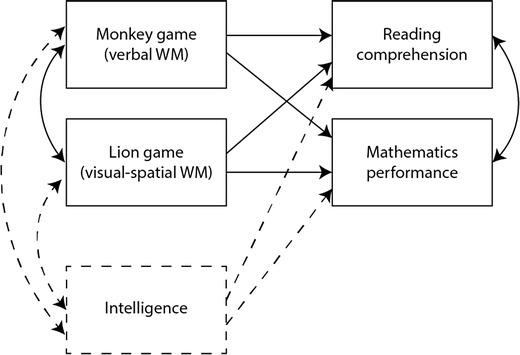
Model 1 (continuous lines only) and Model 2 (also including interrupted lines), either after controlling for grade or when using grade as a grouping factor. WM = working memory

To examine the concurrent and predictive validities of the Monkey game, multilevel multivariate regression analysis was conducted with multiple predictors and two dependent variables, in several steps. A full estimation maximum likelihood (MLR) method was used in Mplus version 7.0, since it is robust to nonnormality and can handle missing data. Although no attempt was made to explain variance at the classroom level, in all models the standard errors were corrected for the nested structure by using an automatic multilevel modeling setup (Stapleton, 2006). That is, applying the Mplus statement “type is complex” ensures that part of the model variance is attributed to between-class variance (i.e., variance in the achievement outcomes existing between classrooms), rather than only to within-classroom variance. First, reading comprehension and mathematics achievement were regressed on the Monkey game and the Lion game. Reading comprehension and mathematics achievement were allowed to covary, as were the two working memory tasks. All variables were controlled for grade. This model is referred to as Model 1. Second, Raven’s SPM scores were added to the model, to examine whether the predictive value of the Monkey game was maintained after controlling for intelligence. This model is referred to as Model 2. Models 1 and 2 are presented in Fig. 2. Since the aim of this study was not to search for the best-fitting model but to examine the strengths of the relationships between variables, both models are saturated models with perfect-fit indices (CFI = 1.000, TLI = 1.000, RMSEA = .000). Third, grade was removed as a control variable, but added as a grouping factor, to explore age-related differences in the concurrent and predictive validities. Steiger’s *Z* (*Z*_H_) was used to test whether differences *within* grades in the dependent standardized estimates (visual–spatial vs. verbal working memory) were statistically significant (Hoerger, 2013; Steiger, 1980), taking into account the covariance between the two working memory tasks. To test whether differences in the independent standardized estimates and covariances *between* the lowest and highest grades were statistically significant, the Fisher *r*-to-*z* transformation was used (Lowry, 2013; Steiger, 1980). When multiple comparisons were made, Holm’s correction was applied to ensure that the chance for a Type I error did not exceed the .05 level. In Holm’s procedure, first the *p* values of the relevant test outcomes are ranked from the smallest to the largest. The smallest outcome *p* value needs to be smaller or equal to *α*/*k* (where *α* = .05 and *k* is the number of tests). The second-smallest *p* value is then compared to *α*/(*k* – 1). This sequence is followed until a corrected *p* value becomes larger than .05. For example, when three comparisons are made, testing at the .05 level, in order to be able to speak of a significant difference, the smallest initial *p* value would need to be ≤.017, the second-smallest would need to be ≤.025, and the final one would need to be ≤.05 (Holm, 1979).

#### Missing data and multiple imputation

Of the 5,203 children in the data set, 36 children (0.7 %) had missing values for all variables. These children probably changed schools before the start of the school year and were removed from the analysis. For the remaining 5,167 children (see Table 1 for the sample characteristics), data at the *unit* level were missing for *n* = 991 (19 %) on the Monkey game, for *n* = 581 (11 %) on the Lion game, for *n* = 595 (11 %) children on mathematics performance, for *n* = 824 (19 %) on reading comprehension, and for *n* = 310 (7 %) on the Raven’s SPM. In total, *n* = 2,954 (57 %) children had complete data for all variables. Additionally, at the *item* level of the Raven’s SPM, data were missing for *n* = 862 (16 %) children on one or more items, due to skipping of items, and for *n* = 69 children (1 %) on one or more items, due to failing to finish the test within the time constraints. The large scale of the study made it unfeasible to keep track of reasons for missingness. However, several reasons were identified as being highly probable. Missing data for the Monkey game, the Lion game, and Raven’s SPM (at the unit level) were most probably due to absence from school during the time of testing. Reading comprehension data were missing in all children from Grade 1, due to the unavailability of the test at mid-school-year. Also, policies differ between schools with regard to whether or not they administer the reading comprehension test in all grades. When reading comprehension or mathematics performance data were missing for individual children, this was most likely because the children changed schools during the study. In general, we expected that the reasons for missing data were mainly due to the absence of children from school (e.g., due to sickness, a dentist visit, or attendance at an official family event) or to a test being unavailable for a certain grade. Thus, it would be plausible to assume that the data were missing at random (Schafer & Graham, 2002). In addition, the pattern of missing data was nonmonotone, and therefore multiple imputation was an appropriate approach to deal with missing data.

**Table 1:** Sample characteristics

	Study 1		Study 2	
	*n*	% Boys	*n*	% Boys
Grade 1	862	51.6	20	65.0
Grade 2	840	53.9	34	47.1
Grade 3	847	51.2	33	57.6
Grade 4	854	48.6	16	68.8
Grade 5	850	49.9	16	56.3
Grade 6	914	49.8	21	42.9
Total	5,167	50.8	140	55.0

To impute the missing data, we constructed the imputation model as follows: All variables from the statistical model (see Fig. 2) were included in the imputation model. Additionally, background variables such as gender, as well as grade and mathematics ability scores from the previous school year, were added to the imputation model. Finally, the reason for missingness [i.e., skipped item(s), failed to finish test and absence during test] was added to the imputation model only for the Raven items. Through the latter procedure, we aimed to strengthen the assumption of data being missing at random. The data had a multilevel structure (students within schools), so this hierarchy should be taken into account while drawing imputations. This ensured congeniality between the imputation and statistical models (Meng, 1994). Congeniality can lead to biased results if the statistical model is more complex than the imputation model and the imputation model ignores important relationships presented in the original data. We therefore included class IDs (as dummy variables) into the imputation model to account for the hierarchical structure of the data (Graham, 2012, p. 135). Five imputed data sets were generated using the MICE package (Van Buuren, 2012; Van Buuren & Groothuis-Oudshoorn, 2011) in R 3.0.2, after investigating the convergence of the MICE algorithm by means of several diagnostic tools such as trace plots. The imputed data set was then analyzed in Mplus (version 7.0), and the parameter estimates and their standard errors were pooled using Rubin’s rules (Rubin, 1987). For comparison, we also performed the analysis on the incomplete data, which implies that about 57 % of the data were used. The distribution of gender in this data set (51.1 % boys) was highly similar to that in the imputed data set.

### Results

Descriptive statistics are presented in Table 2.

**Table 2:** Descriptive statistics for working memory, intelligence, and reading comprehension and mathematics performance for Grades 1 through 6 and for the total sample in Study 1

	Monkey Game (Verbal WM, Mean Proportion Correct Scores)			Lion Game (Visual–Spatial WM, Mean Proportion Correct Scores)			Raven’s SPM (Raw Sum Scores)			Reading Comprehension (Ability Scores)			Mathematics Performance (Ability Scores)		
	*n*	Mean	*SD*	*n*	Mean	*SD*	*n*	Mean	*SD*	*n*	Mean	*SD*	*n*	Mean	*SD*
Grade 1	664	.38	.16	745	.46	.18	810	24.65	7.60	–	–	–	762	34.71	16.07
Grade 2	730	.46	.15	758	.56	.18	799	31.19	8.26	647	14.13	14.91	777	53.23	15.39
Grade 3	644	.51	.14	751	.65	.17	818	35.79	7.65	728	26.27	13.23	722	73.77	15.15
Grade 4	727	.57	.12	744	.71	.15	785	39.97	7.08	654	32.12	13.64	758	86.79	13.67
Grade 5	700	.58	.13	787	.74	.13	793	42.07	6.58	720	46.56	15.35	766	100.90	12.00
Grade 6	711	.62	.13	801	.77	.13	852	44.09	5.97	730	56.26	19.23	787	110.22	14.75
Total	4,176	.52	.16	4,586	.65	.19	4,857	36.33	9.86	3,480	35.60	21.44	4,572	76.75	30.15

WM = working memory, SPM = Standard Progressive Matrices

Cronbach’s alpha for the sum of the first, second, third, and fourth instances within the different levels of the Monkey game was *α* = .89 for the whole sample, ranging from .81 to .88 in the different grades separately. The proportion correct scores for each individual item revealed an internal consistency of *α* = .87 for the whole sample, ranging from .78 to .85 in the different grades separately.

#### ICCs

An ICC of .07 was found for the Monkey game proportion correct scores after controlling for grade at the time of measurement, which indicates that a small proportion of variance was explained by class membership. The design effect for Monkey game scores [1 + (23.69 – 1) × .07 = 2.66] was greater than 2, indicating that clustering needed to be taken into account in the analysis. For the Lion game, reading comprehension, math performance, and intelligence scores, the ICCs were .07, .04, .05, and .07, respectively.

#### Multilevel multivariate regression results

Table 3 presents the standardized estimates of the models tested before (Model 1) and after (Model 2) controlling for intelligence. Analysis with the complete data rendered the same results for both models as the analysis with imputed data. The pooled results from the imputed data sets are reported here.

**Table 3:** Standardized estimates for multivariate regression models before (Model 1) and after (Model 2) controlling for intelligence

		Model 1	Model 2
Regression Coefficients			
Monkey game	ON grade	.51^***^	.51^***^
Lion game	ON grade	.54^***^	.54^***^
Reading comprehension	ON grade	.58^***^	.46^***^
	ON Monkey game	.21^***^	.15^***^
	ON Lion game	.11^***^	.04^***^
	ON Raven	–	.28^***^
Mathematics performance	ON grade	.69^***^	.58^***^
	ON Monkey game	.18^***^	.12^***^
	ON Lion game	.15^***^	.09^***^
	ON Raven	–	.27^***^
Raven	ON grade	–	.66^***^
Correlations			
Monkey game WITH Lion game		.31^***^	.31^***^
Reading WITH Math		.43^***^	.35^***^
Monkey game WITH Raven		–	.34^***^
Lion game WITH Raven		–	.33^***^

^***^ *p* < .001

First, standardized estimates in Model 1 showed that children from higher grades performed significantly better on the Monkey game than did children from lower grades, as well as on the Lion game.

Second, performance on the Monkey game was significantly related to performance on the Lion game after controlling for grade. That is, children with higher scores for verbal working memory also received higher scores for visual–spatial working memory.

Third, performance on the Monkey game was a significant positive predictor of reading comprehension. Performance on the Lion game was also a significant positive predictor of reading comprehension. Comparison of the standardized estimates, however, showed that the predictive value of the Monkey game was stronger that the predictive value of the Lion game for reading comprehension, *Z*_H_ = 6.2, *p* < .001 (Holm’s correction applied).

Fourth, both the Monkey game and the Lion game were significant predictors of mathematics performance. Comparison of the standardized estimates showed that their predictive values were equally strong for mathematics performance, *Z*_H_ = 1.87, *p* = .06.

Fifth, even after controlling for individual differences in intelligence (Model 2), the Monkey game and Lion game remained significant predictors of reading comprehension and mathematics performance, although their standardized estimates became smaller. Moreover, parallel to Model 1, the predictive value of the Monkey game remained stronger than the predictive value of the Lion game for reading comprehension after intelligence was controlled for, *Z*_H_ = 6.8, *p* < .001. Also parallel to Model 1, the predictive values of the Monkey game and the Lion game for mathematics performance were equally strong after controlling for intelligence, *Z*_H_ = 1.85 *p* = .06

Age-related differences in the concurrent and predictive validities of the Monkey game were examined by running Model 2 a second time using grade as a grouping variable, to examine whether the predictive values of the Monkey game differed between age groups. The standardized estimates for each grade separately are presented in Table 4. The results showed that the correlation between the Monkey game and the Lion game increased with grade, as well as the correlation between the two working memory tasks and intelligence scores. Whereas the Lion game was a significant predictor of reading comprehension in Grade 1 only, the Monkey game was a significant predictor of reading comprehension in each grade. As expected, for reading comprehension, the predictive value of the Monkey game was significantly stronger than the predictive value of the Lion game in all grades (*Z* ranges from 2.73 to 4.48, all *p* values were <.001 or <.01 after Holm’s correction was applied), except Grade 1 (*Z* = 2.18, *p* = .03, which is larger than the Holm-corrected *p* value of .007). For mathematics performance, both the Monkey game and the Lion game were significant predictors in each grade except Grade 5, in which only the Monkey game was a significant predictor of mathematics performance. Moreover, the predictive value of the Monkey game seemed fairly stable over grades, whereas the predictive value of the Lion game for mathematics performance diminished in higher grades. In fact, whereas the Monkey game and the Lion game were equally strong predictors for mathematics performance in Grades 1–4 (*Z* ranges from –.49 to 1.37, *p* ranges from .17 to .62), the Monkey game was a stronger predictor than the Lion game in Grades 5 (*Z* = 3.42, *p* < .001) and 6 (*Z* = 5.49, *p* < .001).

**Table 4:** Standardized estimates for the multivariate, multivariable regression model after controlling for intelligence (Model 2) for each grade separately

		Grade 1	Grade 2	Grade 3	Grade 4	Grade 5	Grade 6
Regression coefficients							
Reading comprehension	ON Monkey game	.18^***^	.23^***^	.21^***^	.20^***^	.15^***^	.20^***^
	ON Lion game	.09^**^	.06	.03	.10	.03	.10
	ON Raven	.26^***^	.32^***^	.30^***^	.28^***^	.46^***^	.46^***^
Mathematics performance	ON Monkey game	.19^***^	.23^***^	.22^***^	.20^***^	.16^***^	.26^***^
	ON Lion game	.21^***^	.18^***^	.18^***^	.15^***^	.03	.06^*^
	ON Raven	.40^***^	.37^***^	.38^***^	.40^***^	.47^***^	.42^***^
Correlations							
Monkey game WITH Lion game		.25^***^	.23^***^	.30^***^	.41^***^	.38^***^	.37^***^
Monkey game WITH Raven		.24^***^	.26^***^	.30^***^	.41^***^	.43^***^	.46^***^
Lion game WITH Raven		.26^***^	.23^***^	.31^***^	.37^***^	.46^***^	.44^***^
Reading WITH Math		.41^***^	.32^***^	.36^***^	.44^***^	.41^***^	.33^***^

^***^ *p* < .001, ^**^ *p* < .01, ^*^ *p* < .05

## Study 2

The goal of this study was to further examine the concurrent relationship of the Monkey game with individually administered tester-led working memory tasks. We sought to relate performance on the Monkey game to performance on the self-reliant Lion game, a tester-led verbal working memory task, and a tester-led visual-spatial working memory task. In addition, since the majority of children in Study 2 had also completed the Monkey game in Study 1 (either 1 or 2 years earlier), the stability of performance on the Monkey game was examined.

Since all working memory tasks tap into the domain-general executive component, we expected performance on the Monkey game to be positively related to performance on all three other working memory tasks (Engle, 2002; Engle et al., 1999; Kane et al., 2004). On the basis of the contributions of domain-specific components, we expected the Monkey game to be more strongly related to the verbal tester-led task than the Lion game, whereas we expected the Lion game to be more strongly related to the visual–spatial tester-led task.

### Method

#### Participants

Four of the schools from the large-scale study described under Study 1 participated. The parents of all children received written information about the study, and an active informed consent procedure was used. A total of 140 children received parental consent for participation. The sample characteristics are presented in Table 1.

Of the 140 children, 79 children (58.2 % boys) had also completed the Monkey game 2 years earlier (January–February 2013). In addition, 32 children (46.9 % boys) had completed the Monkey game 1 year earlier, after they had entered first grade (January–February 2014).

#### Measures

##### Working memory

The Monkey game and the Lion game were administered self-reliantly in classrooms. For a description of these games, see the Development and Characteristics of the Monkey Game and Measures sections of Study 1.

In addition, two tester-led computerized working memory tasks from a Dutch version of the Automated Working Memory Assessment battery (AWMA; Alloway, 2007) were individually administered: Word Recall Backward and Odd One Out. Both are computerized tasks in which assessment is led by an assistant in a one-to-one testing situation. In the Word Recall Backward task, a recorded voice names a set of one-syllable words, after which the child is asked to repeat the words verbally in the reverse order. The number of words to remember increases, starting with sets of two words and building up to sets of seven words, after a child correctly recalls four strings of words from the same set in the correct order. When a child incorrectly recalls three strings of words from the same series, task administration is discontinued. The number of correctly recalled words was used for sets in which all stimuli were recalled correctly. The observed scores ranged from 4 to 17.

In the Odd-One-Out task, children are presented with a row of three boxes containing three geometrical shapes on a computer screen. The children are asked to point out the odd shape and to remember its location. Then three new boxes with shapes appear. At the end of each set of items, three empty boxes appear, and the children are asked to point to the consecutive locations of the odd shapes in the correct order. The test starts with sets of one item; after three correct answers of the same length, the sequence is increased by one. When three mistakes are made on sets of the same length, the task is discontinued. The maximum number of items within a set is seven. The numbers of correctly recalled locations were used from sets in which all items were recalled correctly. The observed scores ranged from 3 to 17.

#### Procedure

The measurements took place in January 2015. Children completed the self-reliant Monkey and Lion games, for which teachers received an e-mail containing login information. The tester-led Word Recall Backward and Odd-One-Out tasks were administered individually in a quiet room during school visits by research assistants.

The data from the Monkey game assessments in 2013 and 2014 were extracted from the Study 1 data files.

#### Data analysis

One univariate outlier in Word Recall Backward was found (*Z* score = 3.38) and was removed from the analysis. No multivariate outliers were identified using Mahalanobis distances [*χ*^2^(3) = 16.27]. The normality of the distributions of variables was examined by calculating the standardized skewness and kurtosis indices (statistic divided by standard error). The skewness indices were found to be higher than 3 for the Lion game (–3.44) and the Word Recall Backward task (3.56); the indices for the Monkey game and the Odd-One-Out task were below 3; and the indices for the Monkey game data from 2013 and 2014 were higher than 3 (–3.48 and –3.58, respectively). The kurtosis indices were smaller than 3 for all of the outcome measures.

First, the internal consistency of the scores was calculated in SPSS 22.0 in two different ways, following the methods used by Engle and coworkers (Engle et al., 1999; Kane et al., 2004). More details about these analytic procedures can be found in Study 1.

Second, the associations between the four different working memory tasks were calculated, while controlling for grade. The full-estimation maximum likelihood (MLR) method was used in Mplus version 7.0, since it is robust to nonnormality and can handle missing data. The number of classes in the study was too small to take clustering of the data into account. Since the aim of this study was not to search for the best-fitting model but to examine the strengths of the relationships between variables, a saturated model was used, with perfect-fit indices (CFI = 1.000, TLI = 1.000, RMSEA = .000). Steiger’s Z (*Z*_H_) was used to test whether differences in the standardized estimates were statistically significant (Hoerger, 2013; Steiger, 1980).

Finally, the 1- and 2-year stabilities of the Monkey game were examined by calculating the associations between the Monkey game scores at different time points (between 2013 and 2015, and between 2014 and 2015) using MLR in Mplus. Controlling for grade was only necessary for the first association, since all children in the second analysis were from the same grade (i.e., first grade).

#### Missing data

Three of the 140 students were absent due to illness at the planned time of testing, and therefore they had data missing for the tester-led Odd-One-Out and Word Recall Backward tasks. One student had missing data for the Odd-One-Out task only, due to a technical problem at the time of assessment. Four students had missing data for the Lion game, whereas three students had missing data for the Monkey game. The reasons for missingness for these seven students were unknown but possibly resulted from technical problems (e.g., network problems hampering server connection, and as a result, the functioning of the games).

### Results

Descriptive statistics for Study 2 are presented in Table 5.

**Table 5:** Descriptive statistics for working memory for Grades 1–6 and for the total sample in Study 2

	Monkey Game			Lion Game			AWMA Word Recall Backward			AWMA Odd One Out		
	*n*	Mean	*SD*	*n*	Mean	*SD*	*n*	Mean	*SD*	*n*	Mean	*SD*
Grade 1	20	.45	.14	20	.49	.18	19	5.20	1.32	19	6.68	3.85
Grade 2	34	.47	.14	32	.56	.14	34	6.53	2.14	34	7.62	2.12
Grade 3	33	.54	.15	32	.66	.15	33	7.76	2.57	33	9.61	3.28
Grade 4	16	.60	.14	15	.76	.15	14	8.21	2.36	14	10.64	3.30
Grade 5	16	.60	.11	15	.79	.06	15	9.27	2.15	15	10.40	2.59
Grade 6	20	.61	.12	21	.78	.08	20	9.55	2.74	21	11.89	2.74
Total	139	.53	.15	135	.66	.17	136	7.55	2.66	136	9.24	3.39

AWMA = Automated Working Memory Assessment

Cronbach’s alpha was calculated for the Monkey game as an index of internal consistency. The internal consistency for the sum scores for each of the first, second, third, and fourth instances within the different levels was *α* = .86. The proportion correct scores for each individual item revealed an internal consistency of *α* = .87.

Standardized estimates between the different working memory tasks before and after controlling for grade are presented in Table 6.

**Table 6:** Standardized estimates between working memory assessments: Standardized estimates after controlling for grade in the lower triangle

	Monkey Game	Lion Game	AWMA Word Recall Backward	AWMA Odd One Out
Monkey game^a^	–	.49^***^	.56^***^	.52^***^
Lion Game^a^	.34^**^	–	.44^***^	.57^***^
AWMA Word Recall Backward^a^	.43^***^	.16^**^	–	.47^***^
AWMA Odd-One-Out^a^	.40^***^	.38^***^	.27^**^	–
Grade^b^	.41^***^	.60^***^	.54^***^	.49^***^

^a^Mplus WITH statement, ^b^Mplus ON statement. ^***^ *p* < .001, ^**^ *p* < .01

The Monkey game showed positive relationships with both tester-led working memory tasks (i.e., Word Recall Backward and Odd One Out) and with the self-reliant Lion game. Differences between these standardized estimates were not significant. The relationship between the Monkey game and Word Recall Backward was significantly stronger than the relationship between the Lion game and Word Recall Backward (*Z*_H_ = 2.86, *p* < .01). Although the relationship between the Monkey game and Word Recall Backward seemed to be stronger than the relationship between the Odd-One-Out and Word Recall Backward tasks, this difference was only marginally significant (*Z*_H_ = 1.91, *p* = .06). The Lion game, on the other hand, had a significantly stronger positive relationship with the Odd-One-Out than with the Word Recall Backward task (*Z*_H_ = 2.18, *p* < .05).

The mean Monkey game score in 2013 was .48 (*SD* = .15, *n* = 79), and the mean Monkey game score in 2014 was .46 (*SD* = .15, *n* = 32). The stability of the Monkey game scores over a 2-year period was substantial, with a standardized estimated of .52, *p* < .001 (.44, *p* < .001, after controlling for grade). The association over a 1-year period revealed a standardized estimate of .51, *p* < .001.

## Discussion

The aim of this study was to examine the psychometric properties of the Monkey game for the online self-reliant assessment of verbal working memory in primary school children. The results showed that the Monkey game has good internal consistency and shows good concurrent and predictive validities. In addition, classroom membership influenced working memory performance.

Consistent with the literature, we found significant age effects of verbal working memory scores (Alloway & Alloway, 2010; Alloway et al., 2006). Older children received higher scores on the Monkey game than did younger children, indicating a developmental progression in working memory ability.

The Monkey game showed moderately strong relationships with the other working memory tasks in Studies 1 and 2. Their shared variance might have been due to the contribution of a domain-general executive component, but also might partly have been due to contributions of the domain-specific components. In Study 2, the Monkey game showed a stronger relationship with the tester-led verbal working memory task than the Lion game did, as we expected. The Lion game was more strongly related to the tester-led visual–spatial task than to the tester-led verbal task. There was no difference, however, in the strengths of the relationships between the Monkey game and the tester-led verbal task and the two visual–spatial tasks (tester-led as well as self-reliant assessment), contrary to expectations based on domain-specific overlap between the Monkey game and Word Recall Backward. On the one hand, this finding could be due to the higher similarity in response procedures between the Monkey game and both visual–spatial working memory tasks. This might indicate that the Monkey game’s visual–spatial response format increased its task impurity to a certain extent. On the other hand, we also cannot exclude the possibility that the result may be related to the use of verbal strategies in visual–spatial working memory tasks (Miles, Morgan, Milne, & Morris, 1996), such as verbally recoding the positions of the odd shapes in the Odd-One-Out as “left,” “middle,” or “right.” It has been found that some children use verbal strategies even in mental rotation tasks (Pezaris & Casey, 1991). In Study 1, the strength of the relationship between the Monkey game and the Lion game increased with age. This finding cannot be attributed to differences in variances in working memory, and it is in contrast to our earlier finding that the relationships between several visual–spatial working memory tasks were stronger in younger than in older children (Van de Weijer-Bergsma et al., 2014). These incongruent findings are hard to explain. Should these results be interpreted in light of a validity perspective, or a developmental perspective, or both? From a psychometric point of view, the results may indicate that the validity of the Monkey game is better in older than it is in younger children. However, even if this is the case, several developmental mechanisms might underlie this age-related difference. One developmental mechanism that might explain our results is that as children grow older, performance on both working memory tasks is less influenced by its specific task features and by domain-specific abilities, but increasingly taps into the domain-general component of working memory. In fact, the strengths of the relationships between both working memory tasks and intelligence also increased with age. This is in agreement with earlier findings, from other researchers, that working memory is a more powerful predictor of intelligence in adults (De Ribaupierre & Lecerf, 2006; Kyllonen & Christal, 1990) than in children (De Jonge & De Jong, 1996). Engel de Abreu and colleagues, on the other hand, found that the predictive value of working memory for intelligence remained stable from kindergarten to second grade, whereas the contribution of short-term memory did increase in their study (Engel de Abreu, Conway, & Gathercole, 2010). Indeed, the previous contradictory results regarding the relationship between working memory and intelligence have been interpreted in light of domain-specific (Tillman, Nyberg, & Bohlin, 2008) versus domain-general (Kane et al., 2004) views of working memory. Also, our results may be interpreted in line with the mutualism theory introduced by Van der Maas, Dolan, Grasman, Wicherts, Huizenga, and Raijmakers (2006). According to this theory, scores on the cognitive tasks used in intelligence tests are not correlated at the beginning of development, but they become correlated with development into adulthood due to dynamic interactions between elementary processes: Through direct and indirect positive interactions, each process supports the development of other processes. An example of a reciprocal influence given by Van der Maas et al. was the finding that better short-term memory helps the development of better cognitive strategies, and better strategies make it possible to increase short-term memory efficiency.

Performance on the Monkey showed considerable stability over 1-year and 2-year periods. The stability of the Monkey game seems to be higher than was reported in a longitudinal study by Seigneuric and Ehrlich (2005), for example. They reported correlations between .32 and .33 for the stability of performance on a listening span task over a 1-year period and a 2-year period. Although we have no data available on stability over a shorter period of time (i.e., test–retest reliability over a time period of a number of weeks), these results indicate that the Monkey game provides a reliable measure of verbal working memory over time. Even when children completed the version with a picture response format the first time (i.e., the version for first graders) and the version with a word response format the second time (i.e., the version for second to sixth graders), stability was substantial.

Regarding the predictive value of the Monkey game, we found that verbal working memory scores were predictive of later math achievement as well as of later reading comprehension, which is consistent with the previous literature (Bull et al., 2008; Cain et al., 2004; De Weerdt et al., 2013; Gathercole et al., 2006; Raghubar et al., 2010; Savage et al., 2007; Swanson et al., 2009). The strength of the predictive value of the Monkey game was quite stable over the different grades, which counters the suggestion that the Monkey game may be a more valid measure of working memory in older than in younger children. Consistent with our expectations and the previous literature (Savage et al., 2007), the predictive value of the *verbal* Monkey game for reading comprehension was stronger than the predictive value of the *visual–spatial* Lion game. Moreover, the predictive value of the Monkey game for mathematics achievement increased with age. This finding is consistent with previous studies that have indicated that as children grow older, visual–spatial strategies for solving math problems are replaced with verbal strategies and verbal retrieval (De Smedt et al., 2009; Geary, Hoard, Byrd-Craven, & DeSoto, 2004; Holmes & Adams, 2006; Van der Ven et al., 2013). Since intelligence scores were included in the analyses, we can conclude that the predictive value of the Monkey game lasts above and beyond that of intelligence (De Weerdt et al., 2013; Swanson & Beebe-Frankenberger, 2004). As one might expect, the strength of the relationship between working memory measures and academic achievement decreased after controlling for intelligence. However, since working memory and intelligence measures share a large portion of variance, including intelligence in the analysis may have removed important variance, and consequently may have resulted in an underestimation of the strength of the predictive value of working memory.

Some of the task features of the Monkey game need further consideration. First, with regard to the response format, because the same nine words are used repeatedly and are shown in a 3 × 3 matrix, this could provide an advantage or enhanced learning effect for children who have better encoding skills. On the other hand, this task feature may also increase the difficulty of the task as children advance through it, since previous trials may cause interference with later trials. Also, because children have to translate their response to a visual–spatial format, it is possible that this places greater demands on executive resources than do other backward word span tasks, and it may also increase task impurity (as we discussed earlier). Furthermore, the highly similar results for the Monkey game in Grade 1 and in Grades 2–6, as well as the stability of scores when children from Grade 1 finished the picture version first and the word version 1 year later, indicate that the difference in response formats (pictures vs. words) does not influence the reliability or validity of the task. Second, even in adults, the extent to which a specific task will trigger executive processing is dependent on whether the participant is a novice or an expert in the domain-specific ability that is being targeted (Conway, Kane, Bunting, Hambrick, Wilhelm, & Engle, 2005). Thus, even though we tried to minimize the influence of reading ability in the task, it is possible that individual differences in reading ability influenced performance on the Monkey game. Third, no cutoff rules were applied, indicating that all children finished all trials. Although this might have produced frustration for children who performed poorly on the task, it also increased the sensitivity of the task to individual differences, since information for all trials was included (Conway et al., 2005). Fourth, the verbal instructions in the Monkey game were kept as straightforward as possible. Nevertheless, it is possible that children with less verbal skills had more difficulty understanding the task. It is currently unknown whether such child characteristics (e.g., computer experience or verbal abilities) may confound task performance. In our previous study with the Lion game, however, observations by testing assistants and self-report by children indicated that even children in the youngest age group were able to understand the instructions and play such games self-reliantly (Van de Weijer-Bergsma et al., 2014). Indeed, in the Monkey game, the vast majority of children answered the backward recall practice trials correctly (ranging from 93.3 % in Grade 1 to 99.0 % in Grade 6), indicating that they understood the instructions. In fact, this may actually be an underrepresentation of the number of children who understood the instruction, since there were probably also children who comprehended what they were asked to do but were unable to respond correctly because of working memory difficulties.

To summarize, this study showed that the Monkey game can be used to assess verbal working memory self-reliantly in children within a classroom setting. The Monkey game is a low-cost measure, enabling the inclusion of working memory as a control or predictor variable in large-sample studies. Both the Monkey game and the Lion game can be easily translated into other languages, making cross-cultural comparisons possible without difficulty.^2^ The descriptive statistics of the data from the Monkey game per age group are included in Table 7 for use by other researchers.

**Table 7:** Means and standard deviations for Monkey game scores per age group

		Mean Proportion Correct Score	Absolute Score
Age	*n*	*M* (*SD*)	*M* (*SD*)
6 years	388	.38 (.16)	27.0 (12.1)
7 years	692	.43 (.16)	30.7 (11.7)
8 years	645	.49 (.15)	35.5 (11.5)
9 years	662	.54 (.14)	39.8 (11.4)
10 years	700	.58 (.13)	42.6 (10.6)
11 years	650	.60 (.14)	44.5 (11.6)
12 years^a^	377	.60 (.14)	45.2 (11.9)
13 years^a^	32	.56 (.14)	41.6 (10.7)

^a^Due to the age requirements for school enrollment, students usually leave primary school between 11;6 and 12;6 years of age. The 12-year-old group would thus primarily consists of students with a delayed start and students with a longer educational pathway. The 13-year-old group would consist of students who repeated a class. As a result, the data presented here will be less representative for those particular age groups

## Footnotes

1 Collected by the NWO “Every Child Deserves Differentiated Math Education” Project, Grant Number 411-10-753.

2 Dutch and German versions of the Monkey game and Lion game are available for use by other researchers. Other-language versions of the tasks may also be developed in consultation with the first author. For more information, please contact e.vandeweijer@uu.nl.
